# Immunoglobulin G Locus Events in Soft Tissue Sarcoma Cell Lines

**DOI:** 10.1371/journal.pone.0021276

**Published:** 2011-06-23

**Authors:** Zhengshan Chen, Jing Li, Yanna Xiao, Junjun Zhang, Yingying Zhao, Yuxuan Liu, Changchun Ma, Yamei Qiu, Jin Luo, Guowei Huang, Christine Korteweg, Jiang Gu

**Affiliations:** 1 Department of Pathology, Shantou University Medical College, Shantou, China; 2 Department of Pathology, Peking (Beijing) University Health Science Center, Beijing, China; Brunel University, United Kingdom

## Abstract

Recently immunoglobulins (Igs) have been found to be expressed by cells other than B lymphocytes, including various human carcinoma cells. Sarcomas are derived from mesenchyme, and the knowledge about the occurrence of Ig production in sarcoma cells is very limited. Here we investigated the phenomenon of immunoglobulin G (IgG) expression and its molecular basis in 3 sarcoma cell lines. The mRNA transcripts of IgG heavy chain and kappa light chain were detected by RT-PCR. In addition, the expression of IgG proteins was confirmed by Western blot and immunofluorescence. Immuno-electron microscopy localized IgG to the cell membrane and rough endoplasmic reticulum. The essential enzymes required for gene rearrangement and class switch recombination, and IgG germ-line transcripts were also identified in these sarcoma cells. Chromatin immunoprecipitation results demonstrated histone H3 acetylation of both the recombination activating gene and Ig heavy chain regulatory elements. Collectively, these results confirmed IgG expression in sarcoma cells, the mechanism of which is very similar to that regulating IgG expression in B lymphocytes.

## Introduction

Until recently it was believed that immunoglobulins (Igs) were the characteristic products of only B lymphocytes and plasma cells. However, in the past couple of years several research groups have reported that Igs can also be produced by non-lymphoid lineage cells [1], including human epithelial cancer cells [2], [3], human umbilical endothelial cells [4], human and mouse neurons [5], [6], testicular spermatogenic cells and epididymal epithelial cells [7], and lactating mammary gland epithelial cells [8]. Soft tissue tumors are derived from mesenchyme, and both the clinical behavior and biologic features of sarcomas (malignant soft tissue tumors) differ markedly from those of epithelial neoplasia. Thus far much research has been focused on Ig expression in epithelial cancer cells and the knowledge about Ig expression in soft tissue tumors is quite limited. Recently our group has found that IgG protein was present in a wide variety of sarcoma tissues with IgG protein expression correlating well with proliferation markers and tumor grades [9]. However, whether IgG was actually produced by these sarcoma tumor cells and the molecular basis for IgG expression in soft tissue sarcomas have not been investigated.

The molecular mechanism of variable-diversity-joining (V[D]J) recombination of Ig in B cells has been extensively studied in the past decades [10], [11]. Both the chromatin accessibility of Ig heavy chain (IgH) and the recombination activating gene (RAG) expression were found essential for the initiation of V(D)J recombination. RAG is composed of two enzymes, RAG1 and RAG2, and mice deficient either in RAG1 or RAG2 lost the ability to initiate V(D)J rearrangement [12], [13]. Expression of transfected RAG 1 and 2 in fibroblasts led to rearrangement of artificially accessible recombination substrates but did not result in rearrangement of endogenous antigen receptor loci due to lack of accessibility [14].

In previous studies histone acetylation and germ-line transcription (transcription from unrearranged gene segments) correlated both strongly with an open or an accessible chromatin structure considered to be permissive for V(D)J recombination [15], [16]. In addition, both sense and antisense germ-line transcription were shown to relate well with V(D)J recombination [17], [18] and treatments that activated germ-line gene transcription increased the frequency of Ig gene rearrangement [19], [20]. Several regulatory elements in the RAG locus have been identified, including the proximal enhancer (Ep), the distal enhancer (Ed), and the RAG enhancer (Erag) [21], [22]. The transcriptional regulatory elements of the IgH genes include the V gene–associated proximal promoters, the IgH gene intronic enhancer (Eµ), and the 3′ IgH enhancer (3′ EH) [23]. In B cells certain transcription factors are considered to regulate RAG expression and control the chromatin accessibility by binding to the regulatory elements, thus activating IgH recombination and transcription [24], [25]. A putative RNA editing enzyme, activation-induced cytidine deaminase (AID) is required for both class switch recombination and somatic hypermutation in mouse and human. AID-deficient mice were found unable to produce IgG, IgA, or IgE antibodies [26], [27].

In this study, we investigated IgG locus events in three sarcoma cell lines. We used cell lines instead of primary tumor tissues as the use of cell lines obviated problems of contamination by other cell types, which could arise when analyzing primary tumor tissues given their complex in situ histology with coexisting stroma and lymphocytes. The mRNA sequence of V(D)J recombination of IgG heavy chain was amplified and sequenced. Western blot and immunofluoresence (IF) confirmed the expression of IgG at the protein level. The ultrastructural location of IgG in sarcoma tumor cells was studied with the immuno-electron microscopy (EM) technique. To our knowledge, this was the first time this technique was applied to investigate IgG in cancers. The enzymes essential for IgG expression, including RAG1, RAG2 and AID were also detected in these cell lines. Chromatin immunoprecipitation (ChIP) results showed histone H3 acetylation of both Erag and IgH regulatory elements. These results indicate that the active IgH chromatin status and RAG expression mediate Ig expression in sarcoma cells, the mechanism of which shares many similarities with that controlling Ig expression in B lymphocytes.

## Materials and Methods

### Ethics statement

This study was approved by the ethics committee at Shantou University, China. Written informed consent was obtained from all participants involved in our study.

### Cell culture

The human Ewing's sarcoma cell line A673, osteosarcoma cell line U-2 OS, fibrosarcoma HT1080 and Burkitt lymphoma cell line Raji were obtained from the American Type Culture Collection (ATCC). A673 was cultured in Dulbecco's Modified Eagle's Medium (DMEM, Invitrogen, Carlsbad, CA) with 10% FBS (Hyclone/Thermo Fisher Scientific Inc., Waltham, MA). U-2 OS, HT1080 and Raji were cultured in Roswell Park Memorial Institute (RPMI) 1640 medium (Invitrogen, Carlsbad, CA) with 10% FBS at 37°C in a humidified atmosphere with 5% CO2.

### Isolation of Peripheral Blood Mononuclear Cells (PBMC)

Peripheral blood was obtained from one healthy donor. Mononuclear cells were isolated from 3 ml of peripheral blood using two-step discontinuous Ficoll-Hypaque gradients (Solarbio, Beijing, China). The white gradient layer containing mononuclear cells was collected and washed with 0.01 M PBS, and the isolated mononuclear cells were used immediately for total RNA extraction.

### RNA extraction and RT-PCR

Total RNA was extracted from the cells using Trizol reagent (Invitrogen, Carlsbad, CA) and treated with RNase Free DNase (Invitrogen) to remove genomic DNA. Reverse transcription of total RNA was performed using the Superscript^TM^ III First Strand Synthesis System (Invitrogen, Carlsbad, CA) following the manufacturer's instructions. For the negative control, the reverse transcriptase was omitted in the reaction mixture. Conventional, nested or semi-nested PCR was performed and the primers used in this study are listed in Table 1. The identities of the PCR products were confirmed by DNA sequencing. For IgG1 heavy chain (IGHG1) variable region, VH1, 3, 5 leader and CH1 primers were used in the first round PCR. VH1, 3, 5-FR1 and LJH primers were used in the second round PCR. The PCR products were cloned into a pGM-T vector (Tiangen Biotech, Beijing, China) and sequenced. The V(D)J recombination sequences were aligned with Ig germ-line variable sequences in the Genebank. Barrier tips (Axygen, Union, CA) were used in the whole procedure to exclude cross contamination.

**Table 1:** PCR primers used in this study.

*Gene name*	*RT-PCR primers*	*Primer sequence 5′-3′*	product size (bp)
CD19	The same sense primer	TACTATGGCACTGGCTGCTG	218
	External antisense primer	TGCTCGGGTTTCCATAAGAC	
	Internal antisense primer	CACGTTCCCGTACTGGTTCT	
RAG1	External sense primer	TGGATCTTTACCTGAAGATG	327
	External antisense primer	CTTGGCTTTCCAGAGAGTCC	
	Internal sense primer	CACAGCGTTTTGCTGAGCTC	
	Internal antisense primer	AGCTTGCCTGAGGGTTCATG	
RAG2	External sense primer	TGGAAGCAACATGGGAAATG	193
	External antisense primer	CATCATCTTCATTATAGGTGTC	
	Internal sense primer	TTCTTGGCATACCAGGAGAC	
	Internal antisense primer	CTATTTGCTTCTGCACTG	
AID	External sense primer	GAAGAGGCGTGACAGTGCT	294
	External antisense primer	CGAAATGCGTCTCGTAAGT	
	Internal sense primer	CCTTTTCACTGGACTTTGG	
	Internal antisense primer	TGATGGCTATTTGCACCCC	
IGHG1	External sense primer	ACGGCGTGGAGGTGCATAATG	201
C region	External antisense primer	CGGGAGGCGTGGTCTTGTAGTT	
	Internal sense primer	GACTGGCTGAATGGCAAGGAG	
	Internal antisense primer	GGCGATGTCGCTGGGATAGAA	
Ig κ	Sense primer	TGAGCAAAGCAGACTACGAGA	231
C region	Antisense primer	GGGGTGAGGTGAAAGATGAG	
IGHG1	VH1-leader	CCATGGACTGGACCTGGA	300-330
V region	VH3-leader	CCATGGAGTTTGGGCTGAGC	
	VH5-leader	ATGGGGTCAACCGCCATCCT	
	CH1	ACACCGTCACCGGTTCGG	
	VH1-FR1	CCTCAGTGAAGGTCTCCTGCAAGG	
	VH3-FR1	GGTCCCTGAGACTCTCCTGTGCA	
	VH5-FR1	GAAAAAGCCCGGGGAGTCTCTGAA	
	LJH	TGAGGAGACGGTGACC	
Iγ-Cγ	External sense primer	GGGCTTCCAAGCCAACAGGGCAGGACA	311
	External antisense primer	CAAGCTGCTGGAGGGCACGGT	
	Internal sense primer	GGTGAACCGAGGGGCTTGT	
	Internal antisense primer	CGCTGCTGAGGGAGTAGAGT	
β-actin	Sense primer	TAAAGACCTCTATGCCAACACAG	218
	Antisense primer	CACGATGGAGGGGCCGGACTCATC	
	ChIP PCR primers		
Erag	Sense primer	GCACTGCAAATGGCCTGTGAAC	197
	Antisense primer	TAGAGACCAGAGGGCTTAACATT	
Eµ	Sense primer	CAGCCCTTGTTAATGGACTT	250
	Antisense primer	GGAAAGTTAAATGGGAGTGACC	
3′Cα HS4	Sense primer	TCCAGTCTGAAAAACAAGACC	188
	Antisense primer	ACCTCCCCCAATGCAAATC	
3′Cα HS3	Sense primer	AGGTCTCGACTTAGCACTG	228
	Antisense primer	GGCATGTTTCTCAGAACAGC	

### SDS-PAGE and Western blot

Cell lysates were prepared using cell lysis buffer (Cell Signaling Technology) or RIPA buffer. About 40 µg total cellular protein was separated on 4% to 10% SDS-PAGE gel (IgG under nonreducing conditions and other proteins under reducing conditions). Standard human IgG (0.05 µg/well, Sigma, St. Louis, MO) was used as a positive control and fetal bovine serum (FBS, 10 µl/well, Hyclone) was used as a negative control. The separated proteins were transferred to a polyvinylidene difluoride membrane. Monoclonal mouse anti-human IgG antibody (γ chain specific, Sigma), rabbit anti-human RAG1 (K-20), RAG2 (D-20) or mouse anti-human GAPDH (0411) was used as the primary antibody. RAG1, RAG2 and GAPDH antibodies were purchased from Santa Cruz Biotechnology. After incubation with appropriate secondary antibodies (goat anti-mouse IgG-HRP or goat anti-rabbit IgG-HRP, Santa Cruz Biotechnology), the immunoblots were developed using Super ECL Plus Detection Reagent (Applygen Technologies, Beijing) and exposed to X-ray film according to the manufacturer's protocol.

### Immunofluorescence

Cells were grown on slides and fixed in 4% paraformaldehyde for 15 min at room temperature. The slides were incubated with 0.5% Triton X-100 for 10 min, and blocked for 1 hour in PBS containing 4% bovine serum albumin (BSA). The primary antibody, monoclonal mouse anti-human IgG antibody (γ chain specific, Sigma, St. Louis, MO) or rabbit anti-human κ chain antibody (Zymed Laboratories, South San Francisco, CA) was added and incubated overnight at 4°C. Isotype controls were performed using normal mouse or rabbit IgG at the same concentration as the primary antibodies. The slides were then washed and incubated with goat anti-mouse IgG-FITC (green reaction product) or goat anti-rabbit IgG-TRITC (red reaction product) for 30 min at room temperature. After a final wash, slides were mounted with mounting media with DAPI (Vector Laboratories, Burlingame, CA) and examined under a fluorescence microscope (Carl Zeiss).

### Immuno-electron microscopy

For immuno-EM, the sarcoma cells were fixed in 2% paraformaldehyde with 0.2% glutaraldehyde in 0.1 M PBS (pH 7.4) at 4°C overnight. After dehydration in graded ethanol on ice, the samples were polymerized in LR White resin. A glass knife was used to cut semi-thin sections (1–2 µm), which were stained with toluidine blue, and examined to select areas of interest. Finally, ultra-thin (70–90 nm) sections, cut with a diamond knife, were collected on 200-mesh nickel grids. Dried grids were blocked with 5% BSA in PBST (0.1% Tween 20 in 0.1 M PBS) for 1 hour, followed by incubation with primary antibody overnight at 4°C. The grids were then washed in 0.1 M PBST washing buffer, and then incubated with the secondary antibody for 1 hour. The grids were thoroughly washed in washing buffer and then in distilled water. After air drying, the sections were stained with 5% uranyl acetate, and viewed using a JEOL JEM-1400 transmission electron microscope (TEM) operating at 80 kV. The primary antibody was mouse anti-human IgG antibody (γ chain specific, Sigma) or rabbit anti-human IgG antibody (γ chain specific, Dako Carpinteria, CA, USA). The secondary antibody was 10 nm gold conjugated goat anti-mouse IgG (Sigma, St. Louis, MO, USA) or 20 nm gold conjugated goat anti-rabbit IgG (Abcam, USA).

### Chromatin crosslinking and immunoprecipitation

ChIP was performed as described previously [22]. The rabbit anti-acetyl-histone H3 (06-599, Upstate Biotechnology) was used and normal rabbit IgG (sc-2027, Santa Cruz) was used as a negative control. Immunoprecipitated DNA sequences were analyzed by PCR and the primers are listed in Table 1.

## Results

### Both IgG heavy chain and kappa light chain were expressed in sarcoma cell lines

**Figure 1:**
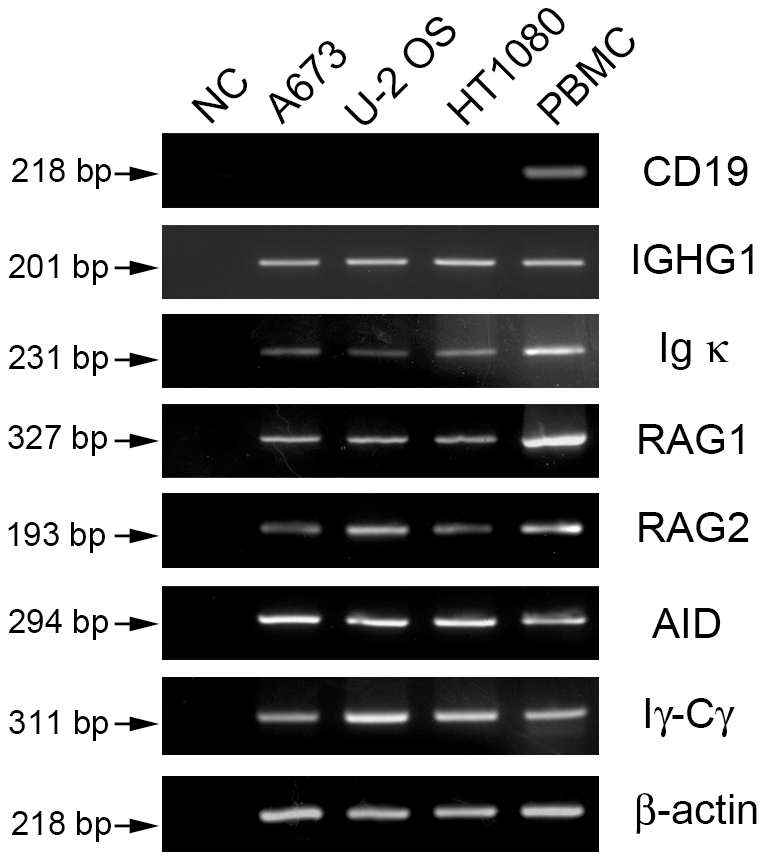
Gene transcripts expression of IGHG1, kappa light chain, RAG1/2, AID and Iγ-Cγ in sarcoma cell lines. β-actin was used as an internal control. PBMC was used as a positive control. DNase treated RNA without adding reverse transcriptase was used as a negative control (NC).

To exclude contamination with B lymphocytes in the sarcoma cell lines, RT-PCR with CD19 primers was performed. CD19 could not be amplified from either Ewing's sarcoma cell line A673, osteosarcoma cell line U-2 OS or fibrosarcoma HT1080 by two rounds of PCR. However it could be detected easily in PBMC (Figure 1). The mRNA of the constant region segments of both IGHG1 and kappa light chains were detected in these sarcoma cell lines (Figure 1).

**Figure 2:**
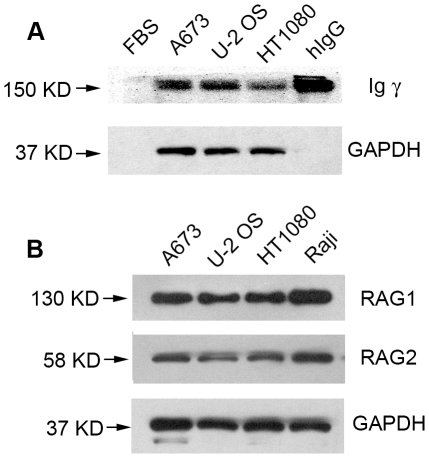
Western blot showing IgG, RAG1 and RAG2 expression in sarcoma cells. A, IgG protein was expressed in sarcoma cells. FBS was used as a negative control and human IgG (hIgG) was used as a positive control. B, RAG1 and RAG2 proteins were both expressed in sarcoma cells. Raji cell was used a positive control. GAPDH was used as an internal control.

IgG proteins were found in all of the three sarcoma cell lines using Western blot assay (Figure 2). The monoclonal mouse anti-human IgG reacted only with human IgG but not with the bovine IgG present in the FBS (Figure 2). This result indicated that IgG proteins were synthesized by the sarcoma cells rather than obtained from the culture medium.

**Figure 3:**
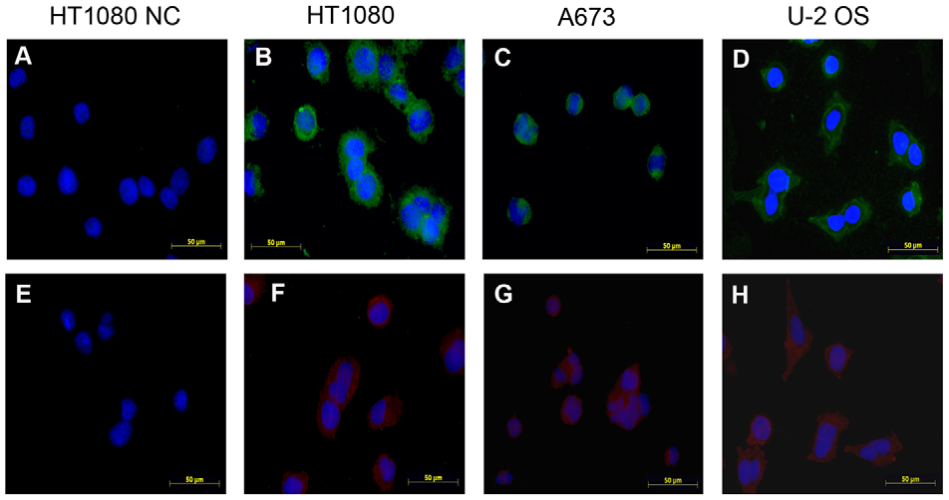
Immunofluorescence showing IgG expression in sarcoma cell lines. A, normal mouse IgG was used instead of the primary antibody (negative control). B to D, the primary antibody was monoclonal mouse anti-human IgG antibody (γ chain specific). A to D, the secondary antibody was goat anti-mouse IgG-FITC. E, normal rabbit IgG was used instead of primary antibody (negative control). F to H, the primary antibody was rabbit anti-human κ chain antibody. E to H, the secondary antibody was goat anti-rabbit IgG-TRITC.

To localize IgG heavy chain and kappa light chain in the sarcoma cells, we performed IF. The result showed that both IgG heavy chain and kappa light chain were expressed in the sarcoma cell lines and that they were located predominantly in the cytoplasm and to a lesser extent on the cell membrane (Figure 3).

### The V(D)J recombination pattern expressed by sarcoma cell lines

Following RT-PCR, the V(D)J recombination sequences were cloned and sequenced. Three clones from each sarcoma cell line were randomly selected for sequencing. The homology of sarcoma derived variable sequences to the germ-line genes at the amino acid level are shown in Table 2. The sequences from all the 9 clones showed potentially functional V region gene recombination because there were no mutations that introduced stop codons into the V region. For the variable region, V_H_5-51 was detected most frequently and could be amplified from A673, U-2 OS and HT1080 cells. The frequency of V_H_5-51 was 4/9 clones. V_H_3-23 could also be amplified from A673 and U-2 OS cells. For the diversity region, D_H_3 was used most frequently and seen in 5/9 clones. For the joining region, J_H_4 usage was most frequent and seen in 5/9 clones (Table 2).

**Table 2:** Deduced amino acid sequences of V_H_-J_H_ transcripts identified in sarcoma cancer lines.

	FWR1	CDR1	FWR2	CDR2	FWR3	CDR3	JH	H %
V3-23D3-3JH4	SLRLSCAASGF***T***F***S***	SYA***MS***	WVRQAPGKG***LE***WVS	***A***ISGSG***GS***TYYADSVKG	RFTISRDNS***KN***TLYLQM***N***SLRAEDTAVYYCAK		HFD***Y***WGQGT***L***VTVSS	
A673 clone 1	------------------***P***--***I***	-----***TG***	-----------------***PQ***------	***G***--------***AA***------------------	----------------***NF***-------------***T-***---------------------------	GGDFGVVT	-----***H***----------***P***---------	92.4%
V3-33D3-3JH4	SLRLSCAASGFTF***S***	***S***YGMH	WVRQAPGKGLEWVA	***VI***WYDGS***NK***YYADSV***K***G	RFT***I***SRDNS***K***NTLYLQMNSLR***A***EDTAVYYCAK		DYWGQGTLVTVSS	
A673 clone 2	----------------------***D***	***D***---------	-----------------------------	***LM***---------***HE***------------***R***--	-----***V***---------***N***-----------------------***V***--------------------	DRGEGLSWFGELF	---------------------------	92.8%
V5-51D2-2JH6	KKPGESLKISC***KG***SGYSF***T***	***S***YW***IG***	WVRQ***M***PGKGLE***W***MG	II***Y***PGDSDTRYSPS***FQ***G	***Q***VTISAD***K***S***I***STAYLQWSSL***K***ASD***T***A***M***Y***Y***CA***R***		LDVWGQGTTVTVSS	
A673 clone 3	------------------***QA***--------***S***	***T***----***LA***	--------***T***------------***L***----	--***F***-------------------***LL***--	***H***----------***T***--***T***--------------------***Q***---- -***A***---***T***---***F***----***K***	MKKGSTSQNS	----------------------------	89.7%
V3-23D3-16JH4	SLRLSCAASGFTFS	SYAMS	WVRQAPGKGLEWVS	***A***I***SGSGGS***TY***Y***ADSVKG	RFTI***S***RDN***SK***NTLYLQM***N***SLR***AE***DTAVYYC***A***K		L***D***YWG***Q***GTLVTVSS	
U-2 OS clone 1	---------------------------	---------	------------------------------	***G***-***IEDSSV***-----***I***------------	------***F***------***HQ***---------------***S***------***VD***--------------***V***--	DYGNCNRGRCYSRP	---***E***-------***R***---------------	88.8%
V5-51D2-2JH6	KKPGESLKISC***KG***SGYSF***T***	***S***YW***IG***	WVRQ***M***PGKGLE***W***MG	II***Y***PGDSDTRYSPS***FQ***G	***Q***VTISAD***K***S***I***STAYLQWSSL***K***ASDTA***M***Y***Y***CA***R***		LDVWGQGTTVTVSS	
U-2 OS clone 2	-------------------***QA***----------***S***	***T***----***LA***	--------***T-***-------------***L***-----	--***F***--------------------***LL***---	***H***-----------***T***--***T***-------------------***Q***-----------***T***---***F***----***K***	MKKGSTSQNS	-----------------------------	90.0%
V5-51D3-22JH5	KKPGESLKISCKGSGYSFT	SYWIG	WVRQMPGKGLEWMG	IIYPGDSDT***R***YSPSFQG	QVT***I***S***A***D***K***SISTAYLQWSSLKASDTAMYYCAR		GFDPWGQGTLVTVSS	
U-2 OS clone 3	------------------------------------	---------	------------------------------	--------------***K***------------	-----***M***-***V***--***N***-----------------------------------------------	LDSTDYRG	-------------------------------	96.9%
V3-74D4-17JH4	SLRLSCAASGFT***F***S	***SY***WMH	WVRQAPGKGLVWVS	***R***IN\***S***DG***SST***TYADSVKG	RFT***I***S***R***DNAKNTLYLQMNSLR***A***EDTAVYYCAR		WGQGTLVTVSS	
HT1080 clone 1	---------------------***L***--	***RS***-------	-----------------------------	***H***---***N***---***DGA***-----------------	----***T***--***K***-------------------------------***E***-------------------	DRNYVAALP	----------------------	90.8%
HT1080 clone 2	---------------------***L***--	***RS***-------	-----------------------------	***H***---***N***---***DGA***-----------------	----***T***--***K***--------------------------------***E***--------------------	DRNYVAALP	----------------------	90.8%
V5-51D3-22JH5	KKPGESLKISCKGSGYSFT	SYWIG	WVRQMPGKGLEWMG	IIYPGDSDT***R***YSPSFQG	QVT***I***S***A***D***K***SISTAYLQWSSLKASDTAMYYCAR		GFDPWGQGTLVTVSS	
HT1080 clone 3	-------------------------------------	---------	------------------------------	---------------***K***--------------	-----***M***-***V***--***N***-----------------------------------------------	LDSTDYRG	-------------------------------	96.9%

Comparisons were made with the closest germ-line V_H_ genes. The bold and italic letter indicates a mutation. H %, homology to the germ-line gene at the nucleotide level.

### The ultrastructural localization of IgG in sarcoma cell lines

**Figure 4:**
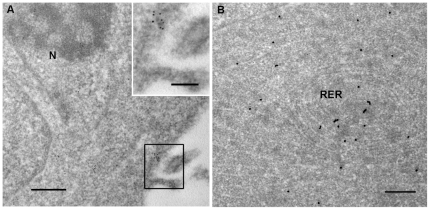
Immuno-EM showing IgG distribution in ultra-thin sections of A673 cells. A, the localization of IgG in A673 cells using 10 nm gold particles. IgG was localized to the membrane of A673 cells. A higher magnification of the boxed area is shown in the insert. Scale bar, 500 nm. Insert scale bar, 250 nm. N, nucleus. B, the localization of IgG in A673 cells using 20 nm gold particles. IgG was localized to the rough endoplasmic reticulum (RER) in the cytoplasm of A673 cells. Scale bar, 500 nm.

To localize IgG in the subcelluar component of sarcoma cells, immuno-EM was performed and the result indicated that IgG was localized to the cell membrane and rough endoplasmic reticulum (RER, Figure 4). RER is the place where proteins are usually synthesized in the cytoplasm. This result provided additional evidence showing that IgG was synthesized in sarcoma cell lines.

### The RAG1, RAG2 and AID enzymes were expressed in sarcoma cell lines

To explore the mechanism of IgG production in sarcoma cells, we investigated the expression of several enzymes, including RAG1, RAG2 and AID. RT-PCR results showed the expression of RAG1, RAG2 and AID in cells of A673, U-2 OS and HT1080 cell lines (Figure 1). The proteins of RAG1 and RAG2 were also detected by Western blot (Figure 2). Germ-line transcription was used as an indicator of chromatin accessibility, and Iγ-Cγ germ-line transcript was detected in these sarcoma cell lines (Figure 1).

### The histone H3 of RAG and IgH regulatory elements were acetylated

**Figure 5:**
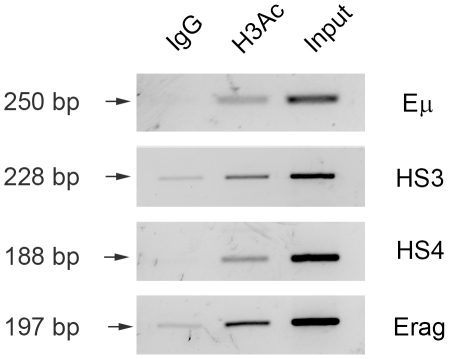
Histone H3 acetylation of the regulatory elements of RAG and IgH gene in A673 cells. HS3, 3′Cα HS3. HS4, 3′Cα HS4. The chromatin precipitated with normal rabbit IgG was used as a negative control. U-2 OS and HT 1080 cells showed similar results (data not shown).

Acetylation of histones H3 and H4 is well known to mark transcriptionally active chromatin. To confirm that the chromatin of RAG and IgH gene was in an open or accessible state, ChIP assay was performed and the result showed that both Erag and IgH (Eµ, 3′Cα HS4 and 3′Cα HS3) contained acetylated histone H3 in the sarcoma cell lines (Figure 5).

## Discussion

In this study we investigated IgG locus events in the mesenchyme derived sarcoma cell lines A673, U-2 OS and HT1080. Both the IgG mRNA segments and proteins were expressed in these cells. V(D)J recombination segments were obtained by RT-PCR and sequenced. Immuno-EM localized IgG specifically to the cell membrane and RER at the ultrastructural level. Iγ-Cγ germ-line transcript was also detected in these sarcoma cell lines. ChIP assay indicated that histone H3 of RAG and IgH regulatory elements were acetylated. These results show that, like epithelial cancer cells [1], the sarcoma cells also have the essential elements for V(D)J recombination and IgG expression. Therefore, IgG production appears to be a common feature of both epithelium and mesenchyme derived tumor cells.

In our RT-PCR experiments, we used barrier tips during the whole procedure to exclude cross contamination. In addition, we always used DNase treated RNA without adding reverse transcriptase as templates of PCR for the negative controls. This kind of negative controls excluded the possibility of genomic DNA contamination and contamination by reagents. Under these rigid quality control conditions, we obtained the V(D)J recombination sequences from the sarcoma cell lines. For the variable region, V_H_5-51 was found in each of the three sarcoma cell lines we used. Interestingly, previous studies have shown that V_H_5-51 was also expressed in cells of breast cancer, lung cancer, colon cancer and oral cancer [28], [29]. V_H_3-23 was found in A673, U-2 OS cells and it has also been found in breast cancer and oral cancer cells [28], [29]. V_H_3-74 was found in HT1080 cells and breast cancer cells [28]. V_H_3-33 was expressed in both A673 and oral cancer cells [29]. For the D_H_ and J_H_ regions, D_H_3 and J_H_4 were used most frequently in the sarcoma cells, which is consistent with the findings in epithelial cancer cells [29]. A recent study on sporadic histiocytic/dendritic cell sarcoma tissues has shown Ig gene rearrangements in sarcoma cells [30]. V_H_3-23 and V_H_5-51 were both amplified and most of the J_H_ gene usages were selected from the J_H_4 family (5/9 cases). Collectively, these results indicate that the V(D)J recombination patterns used by sarcoma cells are similar to those of epithelial cancer cells. Especially V_H_5-51, which was frequently detected in both sarcoma cells and epithelial cancer cells, might be associated with yet unidentified functions in tumors of epithelial and mesenchymal origin.

In B cells, the RAG gene has several regulatory elements to activate its transcription, among which Erag is the most important one. Deletion of this sequence from the mouse germline resulted in a 5-fold to 10-fold decrease in RAG expression and a partial block at the pro-B to pre-B transition [21]. For the IgH gene, Eµ was a strong cis-regulatory element for activating V(D)J recombination in B lymphocytes. Deletion of Eµ caused a significant inhibition of both D_H_ to J_H_ and V_H_ to DJ_H_ rearrangements [31], [32], [33]. The 3′ IgH regulatory region (3′Cα HS4 and 3′Cα HS3) functions to control isotype switching and to influence expression of rearranged V_H_DJ_H_ exons assembled upstream at the J_H_ region [34], [35]. In our study, we have chosen to study the chromatin acetylation status of the most important regulatory elements of RAG and IgH gene, while that of other less important regulatory elements has not been studied.

In the present study we have demonstrated IgG locus events in three sarcoma cell lines, but there are still several questions regarding V(D)J recombination in tumors that need to be answered. For example, in B cells D_H_ to J_H_ recombination occurs in 2 alleles and V_H_ to DJ_H_ recombination occurs only in 1 allele because of allelic exclusion [11]. Whether allelic exclusion also takes place in tumor cells is currently unknown. In B cells several transcription factors, including E2A, EBF, Ikaros, and Pax5 activate RAG expression and V(D)J recombination [23], [24]. Whether these transcription factors are also present in tumor cells has not yet been explored. These questions provide directions for future research. Finding the answers will enrich our knowledge of Ig expression in non-lymphoid lineage cells. Our previous study has shown that IgG expression correlated well with the proliferation markers and tumor grades in sarcoma tissues [9]. The exact role of IgG in tumor progression warrants further investigation.
